# Comparing skill transfer and cognitive style effects across three laparoscopic training modalities: a prospective randomized study in medical students

**DOI:** 10.1007/s00464-025-12511-9

**Published:** 2026-01-13

**Authors:** L. Vradelis, N. Müller, F. Huettl, L. I. Hanke, A. Nedwed, H. Lang, C. Boedecker, T. Huber

**Affiliations:** 1 Department of General, Visceral, and Transplant Surgery, University Medical Center of Johannes Gutenberg-University Mainz, Langenbeckstraße 1, 55131 Mainz, Germany; 2 Institute of Medical Biometry, Epidemiology, and Informatics, University Medical Center of the Johannes Gutenberg-University, Mainz, Germany

**Keywords:** Laparoscopic surgery, Simulation, Surgical education, Laparoscopy, Laparoscopy training

## Abstract

**Background:**

Simulation-based training is an important component of modern surgical education. While virtual reality (VR) simulators, box trainers, and serious games are all used in laparoscopic training, comparative data on their effectiveness, transferability, and the role of individual cognitive learning styles remain limited.

**Methods:**

In this prospective, randomized study, 80 medical students without prior laparoscopic experience were assigned to one of four groups: VR simulator, box trainer, serious game, or control. Each group received four one-hour training sessions. Cognitive style was assessed using the Object-Spatial Imagery and Verbal Questionnaire (OSIVQ), and all participants completed standardized pre- and post-tests on a VR simulator. Performance was evaluated using *z*-scores for efficiency, accuracy, and task completion.

**Results:**

All intervention groups demonstrated significant performance improvements. The VR group showed the greatest gains, particularly in complex tasks such as “Fine Dissection” and “Clip Applying”. Box trainer training led to marked reductions in error rates. The serious game group primarily improved in basic skills but showed limited transfer to complex tasks. Spatial learners outperformed other cognitive styles across all modalities, whereas verbal learners improved significantly only in the VR group.

**Conclusion:**

All three simulation modalities support laparoscopic skill acquisition, but their effectiveness varies by task complexity and cognitive profile. VR training appears to be the most inclusive and effective, particularly for learners with non-spatial cognitive styles. These findings support the integration of cognitive profiling and task-specific modality selection into surgical training.

Laparoscopic surgery has become the standard approach for many general surgical procedures. It offers several advantages for patients, including reduced postoperative pain, faster recovery time, shorter hospital stays, and improved cosmetic outcomes. However, this technique presents a significant challenge for surgeons because of its steep learning curve. Prior research has shown that laparoscopic skill training should begin early in surgical education outside the operating room [1, 2]. Structured training with laparoscopic simulation enables high-quality surgical education in a safe and controlled environment, ideally before the first clinical encounter in an operating room [1, 3].

Studies, including those by Vitish-Sharma et al., have demonstrated that training on both VR simulators and box trainers improves basic laparoscopic skills [4]. This results in a shortened learning curve [5], and the transferability of training effects to the operating room has been well documented [5–8], confirming the clinical utility and effectiveness of simulation training [9–11]. Recently, in a systematic review and meta-analysis of various studies, it has also been demonstrated that technical surgical skills can be transferred from laparoscopic to robotic-assisted surgery (RAS) and vice versa [12].

In addition to established trainers, serious games such as the video game “Underground” for the Wii U console are also available. Despite certain limitations, studies have shown that it is a meaningful and cost-effective complement to traditional training methods [13, 14]. Serious games are defined as digital games that include recreational elements while simultaneously aiming to teach specific procedural or cognitive skills through structured, goal-oriented tasks.

The transferability of skills across various training modalities and into the operating room, especially based on the Fundamentals of Laparoscopic Surgery (FLS) simulator training, has been explored in several studies, revealing a range of results [15–17]. Although there is robust evidence demonstrating the positive effect of structured training on laparoscopic skills, many countries still lack formalized and standardized training programs, and consensus on the optimal training framework remains elusive. Notably, some countries, such as the United States of America, have integrated mandatory laparoscopic training curricula into their national surgical education pathways [18]. In contrast, other countries continue to rely on variable, inconsistent institutional standards. In Germany, recent efforts have focused on developing structured curricula for minimally invasive surgery (MIS) and RAS [19].

Various training systems are available on the market; however, the transferability between these systems has not been thoroughly investigated. This lack of comparative data, combined with the potential influence of individual cognitive learning styles, complicates the selection process for these programs. Given the wide range of available platforms and the high associated costs, institutions face the risk of investing substantial resources into a training system that may not meet residents’ specific educational needs or yield desired outcomes.

The current study aimed to assess the transferability of skills across different training modalities and evaluate the acceptance and usability of each method.

## Materials and methods

**Fig. 1:**
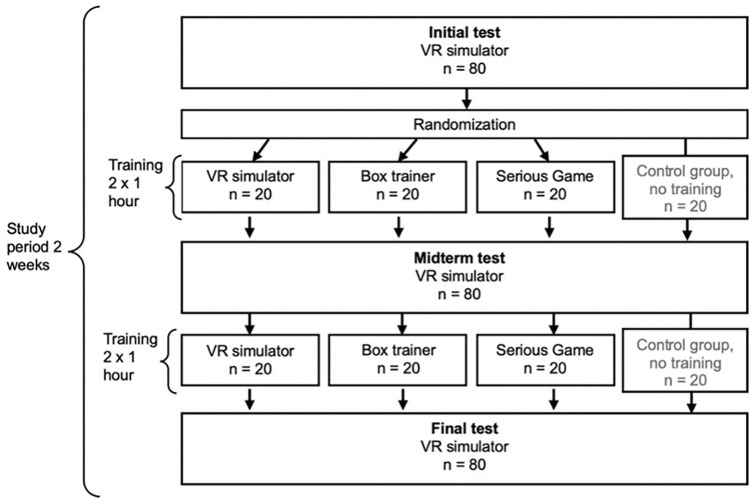
Study design

This prospective preclinical study was conducted at the Department of General, Visceral, and Transplant Surgery at the University Medical Center of Johannes Gutenberg-University Mainz. A total of 80 medical students ranging from the 1st to 8th semester, all of whom had no prior experience in laparoscopic surgery or simulation, were included. After providing informed consent for participation and pseudonymized data storage, the students were assigned to one of four groups: VR simulator (VR), Box trainer (BT), Serious Game (SG), or Control group (CG) (Fig. 1). Before the study, the participants completed questionnaires assessing their demographics, spatial and cognitive abilities, and prior gaming experience. Spatial and cognitive abilities were assessed using the Titmus and mental rotation tests. The Titmus test measures stereoscopic vision, whereas the mental rotation test evaluates the ability to mentally rotate and manipulate three-dimensional objects. Cognitive styles were classified using the Object-Spatial Imagery and Verbal Questionnaire (OSIVQ) [20], identifying participants as object visualizers (VISU), spatial visualizers (SPAT), or verbalizers (VERB). After completion of the study, the usability of each training modality was evaluated by the intervention groups using the System Usability Scale (SUS) [21], and feedback on interest in surgical fields was collected from all participants.

The training protocol consisted of four one-hour sessions, two before and two after the midterm assessment, following a distributed practice structure. Training within each one-hour session was continuous; no scheduled breaks were implemented. Participants trained independently using standardized written instructions, images, and video tutorials provided through a dedicated online course on the institutional learning management system (Moodle™). Although individual engagement with the preparatory materials was not directly monitored, all participants had access to detailed procedural guides and task-specific instructional videos prior to the initial assessment, ensuring baseline familiarity with the fundamental skills. The preparatory content was identical for all participants across groups and consisted of a general introduction to laparoscopic instrument handling and fulcrum effect, as well as task-specific demonstrations corresponding to the VR assessment exercises. Access to these materials was standardized via the institutional LMS, and participants were instructed to review them before the first test session. However, individual usage was not tracked quantitatively, so the extent of informal preparation cannot be precisely determined. Facilitators were present during all training sessions to ensure correct technical setup and adherence to the standardized procedure, but they did not provide individualized coaching or corrective feedback.

Pre-, mid-, and post-tests on the VR simulator consisted of a single standardized repetition of each of the three assessment tasks (“Grasping,” “Fine Dissection,” and “Clip Applying”), in accordance with established VR simulator assessment protocols.

Recruitment efforts included printed notices placed in clinical departments, libraries, and student bulletin boards, complemented by the digital circulation of an informational flyer within student cohort groups through social media platforms.

### Simulators and measurements

- The VR simulator used was the “LapSim” (Surgical Science Sweden AB, Gothenburg, Sweden), equipped with a 27-inch LED monitor, Simballs 4D joysticks and a dual foot switch. Participants in the VR simulator group trained with the exercises "Peg Transfer,” "Lifting and Grasping,” "Pattern Cutting,” "Cutting," and "Seal and Cut,” with performance parameters including time, path length, and angular path. All participants completed the standardized tests on the VR simulator at the beginning, midpoint, and end of the study. These tests involved the tasks of "Grasping,” "Fine Dissection," and "Clip Applying.” Importantly, none of the training modalities practiced these specific VR simulator test tasks. This design was chosen to evaluate the transfer of generic laparoscopic psychomotor skills—such as depth perception, instrument handling, and bimanual coordination—rather than the repetition of identical tasks.
- The box trainer, the “Lübecker Toolbox” (LTB Germany Ltd., Lübeck, Germany), featured a toolbox with an integrated camera and light source and included training modules "Pack your luggage,” "Weaving,” "Nylon twist," and "Triangular cut.” The box trainer's performance was measured using the total time and time penalties for errors [22].
- The serious game used was "Underground" (Grendel Games, Leeuwarden, Netherlands) for the Nintendo Wii U, where participants used laparoscopic-like controllers to navigate complex puzzles and actions, with gameplay incorporating movements similar to those of laparoscopic instruments. For the serious game, progress through game levels and the correlation to time were documented, focusing on skill development and the total levels reached. The levels successfully completed after the four-hour training were documented so that participants could resume from their last completed level in the next training session. Additionally, the times for Levels 2 to 5 and the boss levels in the "Ice" theme were recorded before and after the training. Level 1 of each theme was not documented as it served as an orientation for the graphics and handling of the instruments before starting the game. It has to be stated, that the used serious game and the controllers are no longer available to be purchased.
- The control group did not train and only participated in the testing on the VR simulator.

### Statistics

Performance data from the pre- and post-tests were analyzed using *z*-scores. The *z*-score is a statistical measure that quantifies the number of standard deviations a data point is from the mean of a population. It is calculated using the formula *z* = (*x* − *µ*)/*σ*, where *x* is the raw score, *µ* is the mean of the scores, and *σ* is the standard deviation. A *z*-score of 0 represents the average performance level of the population, while positive *z*-scores indicate above-average performance and negative *z*-scores denote below-average performance [23]. This method was used to assess individual performance relative to the overall group, including specific parameters related to ergonomics and errors. “Economic work” refers to the efficiency of instrument motion and includes the parameters of total path length and angular displacement. Error scores represent the simulator-defined penalties for incorrect actions, including tissue damage, imprecise dissection, instrument collisions, dropped or misplaced clips, and unnecessary instrument movements. “Error reduction” describes the improvement in these penalty-based metrics from pre- to post-test, indicating that participants performed the tasks with fewer or less severe errors after training.

The statistical analysis plan was predefined to use standardized *z*-scores exported by the simulators, as raw absolute values were available, yet the parameters are too different for the individual tasks across all exercises and parameters. Thus, raw score comparisons would lack detail and comparability.

Statistical analysis was conducted using SPSS (SPSS, Version 27, IBM, Armonk, NY), with descriptive statistics and non-parametric tests to compare performance across training modalities, and learning curves reported as means with standard deviations.

## Results

### Study population

The detailed demographic data of all participants are presented in Table 1. The distribution of relevant baseline characteristics was approximately balanced across all intervention and control groups.

**Table 1:** Demographic characteristics of the study participants

Characteristic	Total (*n* = 80)	Intervention group VR (*n* = 20)	Intervention group BT (*n* = 20)	Intervention group SG (*n* = 20)	Control group (*n* = 20)
Gender					
Male *n* (%)	36 (45%)	9 (45%)	10 (50%)	9 (45%)	8 (40%)
Female *n* (%)	44 (55%)	11 (55%)	10 (50%)	11 (55%)	12 (60%)
Age mean (SD)	25.0 (± 3.0)	25.6 (± 2.6)	24.8 (± 3.4)	25.3 (± 3.3)	24.6 (± 3.0)
Handedness					
Right *n* (%)	71 (89%)	19 (95%)	16 (80%)	18 (90%)	18 (90%)
Left *n* (%)	9 (11%)	1 (5%)	4 (20%)	2 (10%)	2 (10%)
Semester mean (SD)	6.3 (± 1.8)	6.1 (± 1.7)	6.9 (± 1.6)	6.3 (± 1.6)	6.1 (± 2.3)
Computer/video games (1 = very often, 5 = never) mean (SD)	3.8 (± 1.4)	3.8 (± 1.3)	4.3 (± 1.2)	3.6 (± 1.5)	3.8 (± 1.4)
Playing musical instruments (1 = very often, 5 = never) *M* (SD)	3.8 (± 1.4)	4.5 (± 1.0)	3.1 (± 1.6)	3.9 (± 1.5)	3.6 (± 1.4)
Interest in surgery (1 = very high, 5 = very low) *M* (SD)	2.1 (± 1.1)	1.9 (± 1.0)	2.5 (± 1.4)	1.8 (± 0.9)	2.2 (± 1.1)
Desire to become a surgeon					
Yes *n* (%)	32 (40%)	9 (45%)	6 (30%)	9 (45%)	8 (40%)
No *n* (%)	16 (20%)	5 (25%)	6 (30%)	2 (10%)	3 (15%)
Undecided *n* (%)	32 (40%)	6 (30%)	8 (40%)	9 (45%)	9 (45%)
Desire to specialize in visceral surgery					
Yes *n* (%)	6 (7.5%)	2 (10%)	1 (5%)	2 (10%)	1 (5%)
No *n* (%)	24 (30%)	6 (30%)	7 (35%)	3 (15%)	8 (40%)
Undecided *n* (%)	50 (62.5%)	12 (60%)	12 (60%)	15 (75%)	11 (55%)
Confidence in assisting in laparoscopic surgery					
Yes *n* (%)	58 (72.5%)	14 (70%)	13 (65%)	19 (95%)	12 (60%)
No *n* (%)	22 (27.5%)	6 (30%)	7 (35%)	1 (5%)	8 (40%)
Assisting in laparoscopic surgery					
Yes *n* (%)	13 (16%)	4 (20%)	3 (15%)	2 (10%)	4 (20%)
No *n* (%)	67 (84%)	16 (80%)	17 (85%)	18 (90%)	16 (80%)
Self-assessment (1 = very good, 5 = very bad) *M* (SD)					
Teamwork *M* (SD)	1.7 (± 0.6)	1.9 (± 0.7)	1.4 (± 0.5)	1.8 (± 0.4)	1.8 (± 0.6)
Communication *M* (SD)	1.8 (± 0.7)	2.1 (± 0.4)	1.5 (± 0.5)	1.8 (± 0.8)	1.9 (± 0.7)
Fine motor skills *M* (SD)	2.6 (± 0.8)	2.6 (± 0.8)	2.8 (± 1.0)	2.5 (± 0.8)	2.4 (± 0.6)
Tests for stereoscopic vision and spatial thinking					
Titmus test (scale 1–9) *M* (SD)	8.6 (± 0.9)	8.6 (± 0.9)	8.6 (± 1.0)	8.7 (± 0.8)	8.6 (± 0.8)
Mental rotation test (scale 1–6) *M* (SD)	3.9 (± 1.1)	3.4 (± 1.3)	3.9 (± 1.1)	4.2 (± 0.8)	4.1 (± 1.1)
Object-Spatial Imagery and Verbal Questionnaire (OSIVQ)					
Object visualizers (VISU) *n*	36	7	7	9	13
Spatial visualizers (SPAT) *n*	24	7	6	7	4
Verbalizers (VERB) *n*	16	6	3	4	3
Not classified (none) *n*	4	0	4	0	0

Age, gender, handedness, and academic background were evenly distributed across all groups

In addition to demographic variables and prior interests, all participants underwent baseline assessments of their stereoscopic vision and spatial reasoning. The Titmus stereotest revealed uniformly high scores across the cohort, with a mean of 8.6 out of 9 (SD =  ± 0.9), suggesting intact depth perception among participants, which is a fundamental prerequisite for laparoscopic tasks. No meaningful differences were observed between the training and control groups.

In contrast, the mental rotation test (MRT) showed moderate spatial reasoning abilities overall, with a mean of 3.9 out of 6 (SD =  ± 1.1) and slightly more variability between groups. The VR group had the lowest average MRT score (*M* = 3.4, SD =  ± 1.3), while the Serious Game (*M* = 4.2, SD =  ± 0.8) and Control (*M* = 4.1, SD =  ± 1.1) groups scored somewhat higher without reaching significance. These findings indicate that, although stereoscopic vision was consistently strong across participants, individual differences in spatial reasoning were present at baseline, potentially influencing training outcomes in modality-specific ways.

### Analysis of pre- and post-test results

**Fig. 2:**
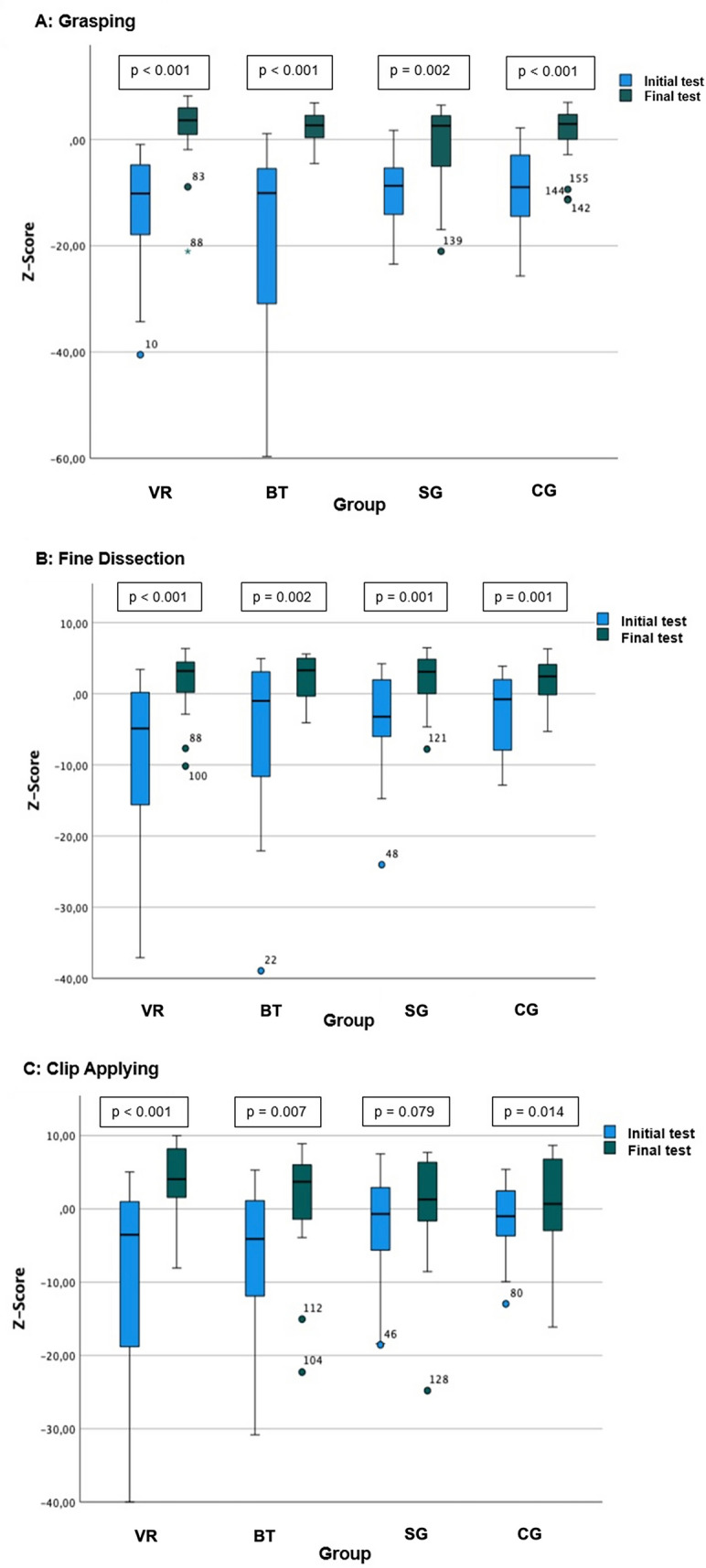
Change in overall *z*-scores from pre- to post-test across all three exercises and all groups. The VR and BT groups showed the steepest gains, and the SG and CG improved more moderately

Across all three exercises, participants in all groups, including the Control group, demonstrated performance improvements from pre- to post-test on the VR simulator (Fig. 2; Tables 2, 3, and 4). Midterm test data were collected to monitor interim progress; however, analyses revealed no significant improvements at this stage compared with the pre-test measurements. Therefore, midterm results were not included in the primary outcome analyses, which focused on the significant changes observed between the initial and final test assessments.

**Table 2:** Pre- and post-test *z*-scores and effect sizes for the "Grasping" exercise

Intervention group		Initial test Md [IQR]	CI [95%]	Final test Md [IQR]	CI [95%]	*p*	Effect size
Total *z*-score							
Group *n* = 80	VR * n* = 20	− 10.2 [− 17.9; − 4.8]	[− 18.6; − 7,5]	3.6 [1.0; 5.9]	[− 1,2; 5,1]	< 0.001	0.8
	BT * n* = 20	− 10.1 [− 30.9; − 5.5]	[− 25.8; − 9.9]	2.7 [0.4; 4.6]	[0.7; 3.7]	< 0.001	0.9
	SG * n* = 20	− 8.7 [− 14.1; − 5.4]	[− 12.9; − 6.2]	2.6 [− 5.0; 4.5]	[− 5.2; 2.6]	0.002	0.7
	CG * n* = 20	− 9.0 [− 14.5; − 2.9]	[− 13.7; − 5.9]	2.9 [0.1; 4.7]	[− 1.6; 3.7]	< 0.001	0.8
*z*-Score economics							
Group *n* = 80	VR * n* = 20	− 4.5 [− 7.7; − 2.5]	[− 8.5; − 3.4]	1.4 [0.4; 2.7]	[− 0.0; 2.3]	< 0.001	0.9
	BT * n* = 20	− 5.6 [− 10.8; − 2.2]	[− 10.1; − 4.1]	1.0 [0.2; 2.4]	[0.3; 1.8]	< 0.001	0.9
	SG * n* = 20	− 4.1 [− 8.0; − 2.3]	[− 6.8; − 3.3]	1.1 [− 1.2; 2.2]	[− 2.4; 1.4]	0.001	0.7
	CG * n* = 20	− 4.2 [− 7.3; − 0.5]	[− 6.8; − 2.4]	1.0 [− 0.2; 2.2]	[− 0.6; 1.5]	< 0.001	0.8
*z*-Score error							
Group *n* = 80	VR * n* = 20	− 2.6 [− 5.0; − 1.3]	[− 8.0; − 1.5]	2.5 [− 0.4; 3.3]	[− 2.0; 2.6]	0.002	0.7
	BT * n* = 20	− 4.3 [− 15.1; − 0.8]	[− 13.7; − 3.8]	1.4 [− 0.3; 2.6]	[− 0.5; 1.9]	< 0.001	0.8
	SG * n* = 20	− 3.4 [− 4.7; − 0.6]	[− 4.9; − 1.4]	0.6 [− 3.5; 1.9]	[− 2.9; 1.0]	0.073	
	CG * n* = 20	− 3.3 [− 5.5; 0.4]	[− 5.3; − 1.6]	1.8 [− 0.2; 2.8]	[− 1.0; 2.1]	< 0.001	0.8

The VR and BT groups demonstrated the largest improvements across all performance domains

*n* number, *Md* Median, *IQR* Interquartile Range, *CI* Confidence Interval, *VR* VR simulator, *BT* Box trainer, *SG* Serious Game, *CG* Control group

**Table 3:** Pre- and post-test *z*-scores and effect sizes for the "Fine Dissection" exercise

Intervention group		Initial test Md [IQR]	CI [95%]	Final test Md [IQR]	CI [95%]	*p*	Effect size
Total *z*-score							
Group *n* = 80	VR * n* = 20	− 4.9 [− 15.6; 0.2]	[− 16.0; − 3.5]	3.2 [0.2; 4.5]	[− 0.4; 3.7]	< 0.001	0.8
	BT * n* = 20	− 1.0 [− 11.6; 3.1]	[− 19.7; 0.2]	3.3 [− 0.3; 5.0]	[0.7; 3.7]	0.002	0.7
	SG * n* = 20	− 3.2 [− 6.0; 2.0]	[− 6.9; − 0.5]	3.1 [0.0; 4.9]	[0.1; 3.8]	0.001	0.7
	CG * n* = 20	− 0.8 [− 7.9; 2.0]	[− 9.5; 0.6]	2.5 [− 0.1; 4.1]	[0.4; 3.5]	0.001	0.7
*z*-Score economics							
Group *n* = 80	VR * n* = 20	− 4.5 [− 8.3; − 0.1]	[− 9.1; − 2.5]	1.9 [1.0; 2.4]	[0.4; 2.3]	< 0.001	0.9
	BT * n* = 20	− 1.4 [− 7.3; 1.2]	[− 11.6; − 0.5]	1.8 [0.6; 2.3]	[0.5; 1.9]	< 0.001	0.8
	SG * n* = 20	− 1.4 [− 3.4; 0.4]	[− 4.1; − 0.5]	1.8 [0.2; 2.3]	[0.1; 2.0]	< 0.001	0.8
	CG * n* = 20	− 0.8 [− 3.5; 0.6]	[− 5.0; 0.1]	0.9 [− 0.1; 2.2]	[0.1; 1.6]	< 0.001	0.8
*z*-Score error							
Group *n* = 80	VR * n* = 20	0.1 [− 4.9; 1.7]	[− 4.5; 0.3]	0.5 [− 0.1; 1.7]	[− 1.0; 1.1]	0.64	
	BT * n* = 20	0.9 [− 1.6; 2.4]	[− 5.8; 1.4]	1.3 [− 0.6; 2.4]	[− 0.4; 1.6]	0.124	
	SG * n* = 20	0.9 [− 1.8; 1.9]	[− 1.9; 1.1]	1.0 [− 0.9; 2.3]	[− 0.3; 1.5]	0.159	
	CG * n* = 20	0.8 [− 2.6; 2.0]	[− 3.2; 1.1]	1.5 [− 0.9; 2.4]	[− 0.0; 1.6]	0.07	

All groups showed significant improvement in overall and economic scores, but error reduction was not significant in any group

*n* number, *Md* Median, *IQR* Interquartile Range, *CI* Confidence Interval, *VR* VR simulator, *BT* Box trainer, *SG* Serious Game, *CG* Control group

**Table 4:** Pre- and post-test *z*-scores and effect sizes for the "Clip Applying" exercise

Intervention group		Initial test Md [IQR]	CI [95%]	Final test Md [IQR]	CI [95%]	*p*	Effect size
Total *z*-score							
Group *n* = 77	VR * n* = 20	− 3.5 [− 18.8; 1.0]	[− 19.8; − 3.0]	4.1 [1.6; 8.2]	[1.7; 6.1]	< 0.001	0.9
	BT * n* = 19	− 4.1 [− 16.6; 1.1]	[− 17.1; − 1.3]	3.7 [− 1.4; 6.0]	[− 2.6; 4.9]	0.007	0.6
	SG * n* = 20	− 0.7 [− 5.6; 2.9]	[− 6.4; 1.0]	1.3 [− 1.6; 6.4]	[− 3.0; 4.2]	0.079	
	CG * n* = 18	− 1.0 [− 3.7; 2.5]	[− 4.4; 0.8]	0.7 [− 3.0; 6.8]	[− 0.8; 4.7]	0.014	0.6
*z*-Score economics							
Group *n* = 77	VR * n* = 20	− 1.2 [− 7.8; 0.9]	[− 7,8; − 0,9]	1.9 [0.5; 3.2]	[0,78; 2,5]	< 0.001	0.8
	BT * n* = 19	− 1.5 [− 4.9; 0.5]	[− 6.2; − 0.6]	1.5 [− 0.7; 2.1]	[− 0.8; 1.9]	0.001	0.7
	SG * n* = 20	0.3 [− 2.3; 1.3]	[− 3.1; 0.6]	1.5 [0.0; 2.4]	[− 1.0; 2.0]	0.037	0.5
	CG * n* = 18	− 0.4 [− 1.5; 0.5]	[− 2.0; 0.2]	0.7 [− 1.1; 2.3]	[− 0.1; 1.8]	0.002	0.7
*z*-Score error							
Group *n* = 77	VR * n* = 20	− 2.2 [− 10.4; 1.6]	[− 10,1; − 0,9]	2.4 [− 0.1; 4.0]	[0.4; 3.0]	< 0.001	0.8
	BT * n* = 19	− 1.2 [− 7.5; 1.5]	[− 9.3; 0.2]	1.8 [− 1.6; 3.1]	[− 1.9; 2.6]	0.064	
	SG * n* = 20	0.1 [− 3.2; 1.8]	[− 2.6; 1.1]	0.9 [− 2.6; 3.6]	[− 2.3; 2.1]	0.433	
	CG * n* = 18	0.6 [− 3.5; 2.8]	[− 2.0; 1.4]	1.1 [− 2.9; 3.8]	[− 0.9; 2.6]	0.231	

The VR group showed the most substantial improvements, particularly in error reduction

*n* number, *Md* Median, *IQR* Interquartile Range, *CI* Confidence Interval, *VR* VR simulator, *BT* Box trainer, *SG* Serious Game, *CG* Control group

#### Grasping

The VR and BT groups showed the most pronounced improvements in all performance domains. Both achieved large effect sizes in overall performance, economic work, and error reduction. While the SG group improved significantly in overall and economic scores, it showed the weakest improvement in error metrics. Notably, the Control group also demonstrated substantial improvement, likely due to repeated task exposure rather than specific training effects (Table 2).

#### Fine dissection

All groups improved significantly in both the overall performance and economic work domains, with the VR group showing the highest effect sizes. However, none of the groups demonstrated statistically significant improvements in error scores, suggesting that this dimension may be more resistant to change over short training intervals (Table 3).

#### Clip applying

The VR group outperformed all others, with large and significant gains in overall performance, economic work, and error scores. In contrast, the SG group failed to reach statistical significance in the overall performance score and showed only modest improvements in the other domains. The Control group showed moderate gains, while the BT group improved across all measures, but to a lesser extent than the VR group (Table 4).

### Subgroup analysis according to thinking styles

The analysis according to thinking styles revealed the following results:

#### Grasping

All thinking style groups (VISU, SPAT, VERB) showed significant improvement across performance domains. The SPAT participants achieved the highest medians and effect sizes across all three domains. VERB participants, while improving, consistently showed the lowest medians and weakest effects on error scores (Table 5).

**Table 5:** Effect sizes and *z*-scores for the "Grasping" exercise by thinking style

Thinking style		Initial test Md [IQR]	CI [95%]	Final test Md [IQR]	CI [95%]	*p*	Effect size
Total *z*-score							
OSIVQ *n* = 76	VISU * n* = 36	− 8.8 [− 18.4; − 4.1]	[− 17.0; − 8.1]	2.9 [− 2.1; 5.0]	[− 1.7; 3.0]	< 0.001	0.8
	SPAT * n* = 24	− 8.0 [− 12.9; − 4.8]	[− 13.0; − 6.2]	3.8 [1.7; 4.7]	[1.5; 4.9]	< 0.001	0.9
	VERB * n* = 16	− 12.6 [− 26.2; − 7.6]	[− 24.2; − 10.0]	0.6 [− 8.2; 4.2]	[− 5.8; 2.0]	0.003	0.8
*z*-Score economics							
OSIVQ *n* = 76	VISU * n* = 36	− 4.0 [− 9.4; − 1.5]	[− 8.0; − 3.9]	0.9 [− 0.4; 2.2]	[− 0.6; 1.3]	< 0.001	0.8
	SPAT * n* = 24	− 3.7 [− 6.5; − 2.1]	[− 6.0; − 2.8]	1.6 [0.8; 2.5]	[0.5; 2.4]	< 0.001	0.9
	VERB * n* = 16	− 5.6 [− 7.2; − 4.3]	[− 9.5; − 4.3]	0.8 [− 1.6; − 1.6]	[− 2.2; 1.3]	0.002	0.8
*z*-Score error							
OSIVQ *n* = 76	VISU * n* = 36	− 2.7 [− 5.5; − 0.2]	[− 7.4; − 2.2]	1.7 [− 1.0; 3.3]	[− 1.3; 1.6]	< 0.001	0.6
	SPAT * n* = 24	− 3.1 [− 4.5; − 1.2]	[− 5.4; − 1.8]	1.6 [0.3; 2.6]	[0.5; 2.1]	< 0.001	0.8
	VERB * n* = 16	− 4.2 [− 10.5; − 1.8]	[− 12.5; − 2.8]	− 0.3 [− 6.3; 2.3]	[− 4.2; 0.7]	0.03	0.5

The SPAT group showed the highest gains across all metrics, and the VERB group showed the least improvement in error reduction

*n* number, *Md* Median, *IQR* Interquartile Range, *CI* Confidence Interval, *OSIVQ* Object-Spatial Imagery and Verbal Questionnaire, *VISU* Visualizers, *SPAT* Spatializers, *VERB* Verbalizers

#### Fine dissection

Participants of all thinking styles demonstrated significant gains in overall and economic performance. SPAT achieved the best results. Error score improvements were observed only in the SPAT and VERB participants, not in the VISU participants (Table 6).

**Table 6:** Effect sizes and *z*-scores for the "Fine Dissection" exercise by thinking style

Thinking style		Initial test Md [IQR]	CI [95%]	Final test Md [IQR]	CI [95%]	*p*	Effect size
Total *z*-score							
OSIVQ *n* = 76	VISU * n* = 36	− 3.6 [− 10.3; 1.3]	[− 13.1; − 2.3]	2.3 [− 1.2; 4.4]	[0.0; 2.5]	< 0.001	0.7
	SPAT * n* = 24	− 1.3 [− 4.9; 2.0]	[− 3.8; 0.1]	4.2 [2.6; 5.5]	[2.7; 4.7]	< 0.001	0.9
	VERB * n* = 16	− 10.0 [− 20.8; − 0.9]	[− 23.6; − 4.9]	1.4 [− 2.1; 3.2]	[− 2.2; 2.7]	0.003	0.8
*z*-Score economics							
OSIVQ *n* = 76	VISU * n* = 36	− 1.9 [− 4.9; 0.6]	[− 7.6; − 1.6]	1.0 [0.2; 2.2]	[0.3; 1.4]	< 0.001	0.8
	SPAT * n* = 24	− 1.0 [− 2.4; 0.9]	[− 2.7; − 0.2]	2.1 [1.7; 2.6]	[1.1; 2.3]	< 0.001	0.9
	VERB * n* = 16	− 5.2 [− 12.2; − 0.8]	[− 12.8; − 3.0]	1.8 [− 1.2; 2.3]	[− 0.6; 1.9]	0.002	0.8
*z*-Score error							
OSIVQ *n* = 76	VISU * n* = 36	0.9 [− 2.9; 2.0]	[− 3.8; 0.2]	0.9 [− 1.3; 1.8]	[− 0.5; 0.8]	0.112	
	SPAT * n* = 24	1.3 [− 1.5; 2.1]	[− 0.5; 1.3]	1.7 [0.8; 2.4]	[1.0; 1.9]	0.023	0.5
	VERB * n* = 16	− 2.5 [− 5.1; 0.9]	[− 7.8; − 0.8]	− 0.9 [− 2.2; 2.0]	[− 2.0; 0.8]	0.015	0.6

SPAT participants again showed superior results, particularly in the economic work scores

*n* number, *Md* Median, *IQR* Interquartile Range, *CI* Confidence Interval, *OSIVQ* Object-Spatial Imagery and Verbal Questionnaire, *VISU* Visualizers, *SPAT* Spatializers, *VERB* Verbalizers

#### Clip applying

The SPAT participants showed the highest performance gains across all domains. The VERB participants exhibited weaker improvements, especially in error scores, where significance was not reached. VISU participants performed better than VERB in medians but achieved lower effect sizes in some domains (Table 7).

**Table 7:** Effect sizes and *z*-scores for the "Clip Applying" exercise by thinking style

Thinking style		Initial test Md [IQR]	CI [95%]	Final test Md [IQR]	CI [95%]	*p*	Effect size
Total *z*-score							
OSIVQ *n* = 73	VISU * n* = 36	− 1.8 [− 6.6; 1.2]	[− 8.9; − 0.8]	2.8 [− 1.2; 6.4]	[− 1.7; 3.5]	0.002	0.5
	SPAT * n* = 24	− 0.8 [− 6.2; 3.4]	[− 8.2; 0.8]	5.2 [1.7; 7.4]	[3.1; 6.0]	< 0.001	0.7
	VERB * n* = 13	− 12.5 [− 20.2; − 0.8]	[− 26.7; − 3.1]	− 1.0 [− 7.1; 3.9]	[− 3.3; 3.4]	0.013	0.7
*z*-Score economics							
OSIVQ *n* = 73	VISU * n* = 36	− 0.3 [− 2.6; 0.7]	[− 3.3; − 0.3]	1.4 [− 0.6; 2.4]	[− 0.6; 1.4]	< 0.001	0.6
	SPAT * n* = 24	0.4 [− 2.7; 1.2]	[− 4.0; 0.2]	2.2 [1.5; 2.7]	[1.5; 2.4]	< 0.001	0.8
	VERB * n* = 13	− 1.9 [− 8.6; 0.2]	[− 9.8; − 0.7]	0.2 [− 2.0; 1.8]	[− 0.8; 1.8]	0.016	0.7
*z*-Score error							
OSIVQ *n* = 73	VISU * n* = 36	− 0.1 [− 2.8; 1.5]	[− 4.6; 0.3]	1.6 [− 1.5; 3.5]	[− 1.3; 1.8]	0.044	0.3
	SPAT * n* = 24	0.7 [− 3.6; 2.6]	[− 3.3; 1.1]	2.7 [0.4; 4.1]	[1.0; 3.2]	0.008	0.5
	VERB * n* = 13	− 5.6 [− 11.6; 1.2]	[− 14.8; − 1.2]	− 2.2 [− 3.4; 1.6]	[− 2.7; 1.3]	0.055	

The SPAT group outperformed the VISU and VERB groups across all performance domains

*n* number, *Md* Median, *IQR* Interquartile Range, *CI* Confidence Interval, *OSIVQ* Object-Spatial Imagery and Verbal Questionnaire, *VISU* Visualizers, *SPAT* Spatializers, *VERB* Verbalizers

## Discussion

This study provides insights into the comparative effectiveness of three different simulation modalities—VR simulator, box trainer, and serious game—for laparoscopic skill acquisition and transfer. In contrast to previous studies that often focus on individual tools or isolated exercises, our investigation simultaneously assessed multiple simulation methods across standardized tasks [6, 24]. With 80 participants, our sample size exceeds that of many prior studies, allowing for more robust statistical interpretation [25, 26].

### Effectiveness of simulation modalities

All training modalities led to significant improvements in laparoscopic performance on the VR simulator. However, the VR group achieved the highest post-test scores, particularly in more complex tasks such as “Fine Dissection” and “Clip Applying.” These results are consistent with the findings of a Cochrane review by Nagendran et al., which suggests that VR training can enhance operative performance and reduce operating time—especially compared to no training, and to some extent, in comparison to box trainer training [27]. The box trainer also led to significant improvements, particularly in terms of precision and error reduction, confirming its effectiveness in fostering core laparoscopic competence. This aligns with evidence from two Cochrane reviews [5, 28], which showed that box trainer-based training significantly improves task time, accuracy, and error rates—even in trainees with no or limited prior laparoscopic experience. Furthermore, it is consistent with the results of a randomized controlled trial by Thomaschewski et al., who demonstrated that box trainer-based training with the “Lübecker Toolbox Curriculum” significantly enhanced real-world operating room (OR) performance in laparoscopic cholecystectomy among novice residents [29]. In contrast, the serious game group showed weaker performance improvements, especially in the “Clip Applying” task and in error metrics, suggesting that while games may offer motivational and ergonomic benefits, their transferability to complex surgical tasks remains limited. This echoes the existing literature on serious games in surgical education, which underscores their role as a fun and cost-effective supplement rather than a substitute for structured training [13, 14]. It has to be stated that the used serious game and the controllers are no longer available for laparoscopic training.

An interesting observation was the greater variability in the initial scores within the VR and Toolbox groups. While this range may reflect individual differences in baseline abilities or learning trajectories, the lack of significant interim improvements suggests that measurable gains occurred primarily later in the intervention period. Although midterm assessments were conducted after two training sessions to monitor interim progress, statistical analysis revealed no significant improvements compared to pre-test performance. This finding may indicate that a threshold of repeated exposure or task familiarity is necessary before measurable learning effects emerge, particularly in complex psychomotor domains. As such, short-term training benchmarks may fail to capture early cognitive adaptation or motor planning processes that only translate into performance gains after extensive repetition. Future studies should explore more frequent interim assessments, adaptive pacing strategies, and training durations to better characterize the temporal dynamics of laparoscopic skill acquisition. This could enable a more profound understanding of the minimal and optimal training durations for future research projects, as well as to reach clinical proficiency.

### Cognitive styles and personalized training

A novel and distinctive aspect of our study is the analysis of individual cognitive styles (VISU, SPAT, and VERB). Across all exercises, SPAT consistently outperformed other styles in terms of *z*-scores, error reduction, and efficiency. This is in line with research showing that spatial thinking styles predict superior performance in laparoscopic skill training on box trainers and are associated with increased efficiency and accuracy in simulated surgical tasks [30].

Interestingly, VERB showed marked improvement only in the VR group. This may indicate that VR simulators offer structured guidance and immersive feedback that benefit learners with non-spatial cognitive profiles.

While the VR modality was the most effective across the cohort, these findings suggest that it may be particularly valuable for leveling cognitive differences among learners. Rather than simply outperforming other methods, VR training appears to be more inclusive, supporting a broader range of cognitive styles. This highlights the potential of personalized training strategies that consider not only modality but also learner characteristics to optimize both effectiveness and equity in surgical education.

In addition to cognitive styles, baseline assessments demonstrated uniformly high stereoscopic vision but considerable variability in spatial reasoning among the participants. While all individuals possessed sufficient depth perception for laparoscopic tasks, differences in mental rotation ability may have significantly affected training outcomes. These findings underscore the importance of integrating visuospatial ability profiling into the surgical education curriculum. Tailored training interventions, particularly those leveraging immersive VR technologies, may facilitate more equitable and efficient skill acquisition for learners with lower spatial aptitude. Future investigations should evaluate the long-term impact of such personalized approaches on both skill retention and their transfer to clinical practice.

### Integration of current evidence

The findings of our study are reinforced by systematic reviews by Gurusamy et al. and a randomized controlled trial by Thomaschewski et al.:

- VR training significantly improves operative skills and reduces task times; however, clinical outcomes remain understudied [27].
- Box trainer training leads to significantly better technical performance compared to no training or standard apprenticeship alone, particularly among trainees without prior experience [5, 28].
- Both modalities are considered effective and cost-efficient tools for early phase laparoscopic education, and structured integration into the surgical curriculum is recommended [29].
- Our results expand on this by providing direct comparative evidence across training types in a uniform test environment (VR simulator), while also highlighting individual learner characteristics as mediators of the training success.

### Limitations of the study

Despite the positive findings, this study has a few limitations.

First, the total training duration was limited to four hours. This restricts conclusions regarding long-term retention and skill transferability to real-world surgical settings. It is possible that the improvements observed would be more pronounced with longer training. However, they may also not be sustained without continued practice. Therefore, future studies should incorporate longitudinal follow-ups and evaluate surgical performance in actual clinical environments. Moreover, the observed improvements reflect immediate post-training performance rather than durable skill acquisition. Short-term gains on a simulator are known to capture early familiarization and task-specific adaptation, but they do not necessarily indicate consolidation of higher-order surgical skills or retention over time. Given the limited training duration, our study cannot differentiate between transient performance enhancement and sustainable skill development. Longer and repeated follow-up assessments would be required to determine whether the observed improvements persist, plateau, or diverge between modalities.

Second, the training followed a time-based structure, meaning that all participants received the same duration of training regardless of performance progression. Our study was not designed to compare proficiency-based training with time-based training models.

Third, all post-training assessments were performed using a VR simulator, which, although it offered standardized conditions, may have slightly favored the VR training group. However, transferability of the VR simulator performance to the operating room has been described before which makes the simulator an acceptable tool for training progression measurement. The exclusive use of VR for pre-, mid-, and post-testing may have amplified a platform-specific familiarity advantage for participants in the VR training group, beyond true cross-modal transfer. Although VR-to-OR transfer has been reported, this does not eliminate the possibility that VR-trained participants were advantaged by being assessed on the same platform. Box trainers have also demonstrated transfer to operative performance, and our study design cannot distinguish platform familiarity from broader skill acquisition effects. In addition, the Control group also showed substantial improvement from pre- to post-test. Repeated exposure to the same assessment tasks should be considered a form of unsupervised skill rehearsal rather than an inert control condition. The Control group’s improvement therefore likely reflects genuine learning effects driven by task repetition, which may lead to an underestimation of the true differences between structured and unstructured training modalities.

Fourth, the absence of clinical or intraoperative performance data limits the generalizability of our findings, although skill transfer to the operating room has been proven before. Future studies should additionally include direct assessments of clinical performance.

Moreover, while our analysis of cognitive styles provided valuable insights into individual differences in simulator-based performance, the reliance on self-report instruments such as the OSIVQ may limit the precision and objectivity of cognitive profiling. Moreover, although our findings point to the potential benefits of tailoring training to individual learning styles, the study was not planned to investigate thinking styles primarily. Therefore, future research should explore the effectiveness of such individualized training approaches.

## Conclusion

Minimally invasive surgery requires the early acquisition of laparoscopic skills, and simulation-based training offers a safe and resource-efficient alternative to traditional intraoperative learning. While the transferability of simulation-acquired skills to real-life surgery is well supported in the literature, standardized and personalized training protocols are still lacking, highlighting the need for further research.

Our findings demonstrate that both box trainer and serious game training can improve performance on a VR simulator, although the serious game was less effective, particularly for complex tasks. Spatial thinkers consistently showed strong improvements across all modalities, whereas verbalizers may have benefited most from the structured and feedback-rich environment of the VR simulator. These results underscore the importance of aligning simulation-based training with individual cognitive styles to maximize the learning outcomes.

Basic laparoscopic skills appear to be transferable across different simulation formats. However, the acquisition of advanced techniques requires direct practice on high-fidelity platforms. While all three training modalities showed effectiveness to varying degrees, the ability of serious games to support transfer to more complex tasks and other simulation environments remains limited and warrants further research. Future studies should examine long-term skill retention, define optimal training durations, and explore the advantages of proficiency-based curricula tailored to diverse learner profiles.

## Abbreviations

BT: Box trainer
CG: Control group
FLS: Fundamentals of Laparoscopic Surgery
MRT: Mental rotation test
OR: Operating room
OSIVQ: Object-Spatial Imagery and Verbal Questionnaire
RAS: Robotic-assisted surgery
SD: Standard deviation
SG: Serious game
SPAT: Spatial visualizers
SPSS: Statistical Package for the Social Sciences
SUS: System Usability Scale
VERB: Verbalizers
VISU: Visualizers
VR: Virtual reality
